# Loneliness as a predictor of outcomes in mental disorders among people who have experienced a mental health crisis: a 4-month prospective study

**DOI:** 10.1186/s12888-020-02665-2

**Published:** 2020-05-20

**Authors:** Jingyi Wang, Brynmor Lloyd-Evans, Louise Marston, Farhana Mann, Ruimin Ma, Sonia Johnson

**Affiliations:** 1 Division of Psychiatry, University College London, Maple House, 149 Tottenham Court Road, London, W1T 7NF UK; 2 Department of Social Medicine, School of Public Health, Fudan University, Shanghai, 200032 China; 3 Research Department of Primary Care and Population Health, University College London, UCL Medical School, Rowland Hill Street, London, NW3 2PF UK; 4 Camden and Islington NHS Foundation Trust, St Pancras Hospital, 4 St Pancras Way, London, NW1 0PE UK

**Keywords:** Loneliness, Mental disorders, Symptom severity, Affective symptoms, Recovery, Quality of life

## Abstract

**Background:**

Loneliness has not until recently been a prominent focus in research on outcomes of mental illness. The aim of this study was to determine whether loneliness at baseline predicts poor outcomes at 4-month follow-up for individuals who have experienced mental health crises. The outcomes in this study included overall symptom severity, affective symptoms, self-rated recovery and health-related quality of life.

**Methods:**

Our study reports a secondary analysis of data from a randomised controlled trial. The sample (*n* = 399) was taken from patients who received treatment from community crisis services. Respondents (*n* = 310) completed the follow-up measurement 4 months after baseline. Loneliness at baseline was assessed using an eight-item UCLA Loneliness Scale. The four mental health outcomes were measured at both baseline and follow-up. Two scales (or part thereof) assessed objective social isolation and neighbourhood social capital at baseline. Regression analyses were conducted to investigate longitudinal associations between loneliness at baseline and mental health outcomes at follow-up.

**Results:**

Loneliness at baseline was associated with all four mental health outcomes at 4-month follow-up, adjusting for psychosocial, socio-demographic and clinical characteristics. A one-point higher loneliness score was associated with 0.74-point (95% CI 0.45, 1.02) and 0.34-point (95% CI 0.21, 0.47) increase in overall symptom severity score and affective symptoms score respectively, and with 1.08-point (95% CI -1.45, − 0.71) and 1.27-point (95% CI -1.79, − 0.75) decrease in self-rated recovery score and health-related quality of life score respectively. Loneliness was a better predictor of clinical outcomes than objective social isolation and social capital, even though the associations with clinical outcomes were reduced and no longer statistically significant following adjustment for their baseline values. A significant association with quality of life persisted after adjustment for its baseline score.

**Conclusions:**

Greater loneliness at baseline predicted poorer health-related quality of life at follow-up. There were cross-sectional associations between loneliness and clinical outcomes, but their longitudinal relationship cannot be confirmed. Further research is needed to clearly establish their underpinning pathways. Reducing loneliness may be a promising target to improve recovery for mental health community crisis service users.

## Background

Mental health problems often have a chronic or episodic course which could give rise to impairment of all life domains. Among potential prognostic factors for mental health problems, social indicators both at societal and individual level have received increasing interest in research. Schwarzbach and colleagues [1] systematically reviewed evidence on whether various subjective and objective assessments of social relations are related to depression in later life, concluding that subjective measures of quality of social relationships appeared more closely related than measures of quantitative aspects of social relationships. Loneliness is a noteworthy example of a potentially important subjective social influence on mental health [2]. It can be construed as a painful experience that happens when there is a subjective difference between the desired and actual social interaction [3–5]. However, objective social isolation refers to observable social contacts – having few or no meaningful relationships with others [6, 7]. Social capital can be defined as “a series of resources that individuals earn as a result of their membership in social networks, and the features of those networks that facilitate individual or collective actions” [8, 9]. Objective social isolation and social capital are often objectively measured according to quantitative aspects of social relationships or resources, and thus different from loneliness [10].

The impact of loneliness on physical health has been studied a lot [11, 12]. For example, two meta-analytic reviews reported that loneliness is related to greater mortality rates, and that its impact is comparable with some well-established risk factors such as obesity, smoking, and physical inactivity [13, 14]. Loneliness is also predictive of development of coronary heart disease and stroke [15], and of faster rate of growth in systolic blood pressure [16], and development of fatigue and pain [17] in longitudinal studies. However, loneliness has not until recently been a prominent focus in research on outcomes of mental illness. Associations have been found with psychosis [18, 19], depression [20] and increases in depressive symptoms [21], personality disorders [22], suicide [23], cognitive decline [24], Alzheimer’s Disease [25], and impaired executive control [26]. However, there is not much longitudinal evidence on relationships between loneliness and mental illness, and most of the existing studies are cross-sectional research, from which causal relationships cannot be inferred. In a recent systematic review of loneliness and perceived social support and outcomes of mental health problems, loneliness has been investigated much less than perceived social support with only two prospective studies retrieved for the review including loneliness as an independent variable [27].

Thus our study addresses a significant gap in the literature in investigating whether loneliness is a longitudinal predictor of recovery following a mental health crisis. Crisis resolution team (CRT) users are clinically a mixed group of people with a variety of psychiatric diagnoses all of whom are immediately post mental health crises in which inpatient admission would otherwise be required [28]. As CRT users have just suffered mental health crises, predictors of recovery are particularly relevant for them. We hypothesise that greater loneliness at baseline will predict more severe overall symptoms, more severe affective symptoms, poorer self-rated recovery, and poorer health-related quality of life at 4-month follow-up in a cohort who have been using CRT services following a crisis.

## Methods

### Study design and participants

The sample was recruited to take part in the CORE study (CRT Optimisation and RElapse prevention) which is a randomised controlled trial of peer-supported self-management intervention. The study protocol paper gives a full account of methods [29] and the outcomes of the trial have been reported [30]. The present study reports a secondary analysis of data from this trial and is part of the work conducted for the first author’s PhD thesis [31]. Participants were interviewed initially in 2014–15 as close as possible to the point of discharge from the CRT, i.e. once they had received treatment from the CRT for at least a week and within 1 month of CRT discharge, and re-interviewed 4 months after recruitment. Face-to-face interviews were conducted at participants’ accommodation, or National Health Service (NHS) or university premises. The CORE target population was CRT users who had received services for no less than a week from a participating CRT in one of the six NHS Trusts in the UK and who could give written informed consent. We excluded service users if they presented a high level of risk to other people, did not live in the catchment area, could not understand English, or were discharged from the CRTs more than 1 month. The sample in this study only involved participants in the main trial. The individuals recruited for the pilot CORE study were not included as loneliness data were not collected from any of these participants. For the main trial, the researchers assessed 3054 CRT users for eligibility at baseline. Of these, 1697 met the inclusion criteria and were contacted for participation. Four hundred and one (23.6%) participants were enrolled and finished the baseline assessment. The major reasons for the eligible CRT users not participating in the study were declining to take part (*n* = 539), not being contactable (*n* = 317), and not completing the assessment in a month of being discharged from the crisis team (*n* = 319). Additionally, two participants were excluded as they withdrew their consent to the use of their data, which gave rise to the study sample of 399 participants available for follow-up interview (Additional file 1: Fig. S1). The London Camden and Islington Research Ethics Committee approved the CORE study protocol. Written informed consent was provided by all the participants.

### Measures

The measures used for the analyses in this paper were described here.

### Outcomes of mental health problems

The four study outcomes, overall symptom severity, affective symptoms, self-rated recovery, and health-related quality of life, were assessed at baseline and at 4-month follow-up.

#### Overall symptom severity

The 24-item Brief Psychiatric Rating Scale (BPRS 4.0) [32] was used to assess participants’ overall symptom severity. The maximum score was 168 based on a 7-point scale. Higher scores indicate more severe overall symptoms. The BPRS has shown good to excellent inter-rater reliability in existing studies with intraclass coefficients from 0.62 to 0.97 [32–35].

#### Affective symptoms

The affective symptom subscale score was derived from the four items of BPRS (anxiety, depression, suicidality, and guilt) in accordance with the suggestions of a review which meta-analysed previous factor analyses of the 24-item BPRS [36]. The four items were summed to a score indicating affective symptoms.

#### Self-rated recovery

The 22-item Questionnaire on the Process of Recovery (QPR) [37] was used to assess self-rated recovery. Each item consists of a declarative statement (e.g. “I feel better about myself”). Participants specified their degree of agreement in five points that ranges from 0 (disagree strongly) to 4 (Agree strongly). Higher total scores indicate better recovery. The QPR possesses satisfactory internal consistency, construct validity and reliability [37].

#### Health-related quality of life

A vertical, 0–100-point visual analogue scale (VAS) as part of the Euroqol 5-Dimension Health Questionnaire (EQ-5D) [38] was used to assess health-related quality of life. The VAS rated the overall health status with endpoints of best state set at 100 and worst state set at 0. EQ-5D is a psychometrically sound tool [39, 40] and currently is being widely used in different countries by clinical researchers in a variety of clinical areas [38].

### Loneliness

Loneliness was assessed at baseline using an eight-item short-form measure of the University of California at Los Angeles (UCLA) Loneliness Scale (ULS-8) [41] which was reported to have a high level of validity and reliability [41–43]. This questionnaire evaluates feelings of loneliness in life in terms of its frequency and intensity. The items were answered on 4-point scales (never, rarely, sometimes, and always). The maximum score was 32 with higher scores indicative of greater loneliness.

### Psychosocial variables

Social network size was measured at baseline. The sum score of items 1 and 4 (How many relatives/friends do you see or hear from at least once a month) of the Lubben Social Network Scale (LSNS-6) [44] was used, with higher scores indicating larger social network size. These two items provide a measure of objective social isolation. The other four items were excluded as they measure subjective quality of social contact and considerably overlap with the loneliness scale.

Perceived neighbourhood social capital was measured at baseline using the Health and Lifestyles Survey Social Capital Questionnaire [45], with higher scores indicating greater social capital.

### Potential confounders

The potential confounders were measured at baseline. Socio-demographic information was collected, including age, sex, ethnicity, whether born in the UK, housing and living situation, contact with children, education, and employment. Number of psychiatric inpatient hospitalisations, number of years since first contact with mental health services, and participants’ clinical diagnoses were also collected.

### Statistical analysis

#### Missing data

Participants who were followed-up at the 4-month point were included in the analyses. The four outcome variables of overall symptom severity (BPRS), affective symptoms (affective symptom subscale of BPRS), self-rated recovery (QPR) and health-related quality of life (EQ VAS) at follow-up had missing items for 2.9, 1.6, 2.6 and 1.9% of participants respectively. For the four variables at baseline, the corresponding percentages were 2.0, 1.0, 2.5 and 0.8%, respectively. All participants completed the loneliness scale (ULS-8) and 2.0% of participants had 1–2 items missing. For the other variables the percentages of participants missing one, more, or all items ranged from 0.3–1.8%. Because of the low percentage of missing data, case mean substitution was conducted. The missing data point was replaced with the participant’s mean on the basis of the items that were collected for the participant [46]. We only applied this approach for loneliness scale, BPRS subscales, QPR, and social capital variables, because it is valid to use this method when all items indicate the same construct or concept in a scale [46] and when they are comparable and roughly interchangeable [47].

#### Descriptive statistics

Participants who completed follow-up assessment were compared with those who did not to examine potential bias. The baseline variables between the two groups were compared using independent sample t-tests (for continuous variables) and chi-square tests (for categorical variables). Descriptive statistics were carried out for dependent variables at follow-up. The four dependent variables at baseline and at follow-up were compared respectively by paired sample t-tests. Cohen’s d was used to calculate the effect size of their change.

#### Multivariable regression models

The association between loneliness at baseline and overall symptom severity at follow-up was tested using linear regression, with follow-up BPRS total score as dependent variable and baseline ULS-8 total score as independent variable. Then baseline social network size and social capital were added to the second model in order to test whether the observed association was independent of psychosocial variables. In the third step, we adjusted for baseline factors which were associated with both dependent and independent variables in univariate analyses with *p* < 0.25 (see Additional file 1: Tables S2 and S3). Finally, baseline BPRS total score was added to the fourth model. We repeated the analyses with BPRS affective symptom subscale score, QPR total score, EQ VAS score as dependent variables, respectively. All analyses were performed using Stata version 12.1.

## Results

### Lost to follow-up

Of the 399 baseline respondents, 89 were lost to follow-up as they declined, could not be contacted, deceased, were unwell, or their risk levels changed. Therefore, 310 (77.7%) respondents completed the 4-month follow-up assessment and were included in this paper (Additional file 1: Fig. S1). Compared to non-completers, completers were more likely to have independent accommodation (*p* = 0.01), and to have regular employment or voluntary, protected or sheltered work (*p* = 0.01). However, no other measures showed statistically significant differences between the two groups. A comparison of baseline variables between respondents who participated at follow-up and those who did not is given in Table 1.

**Table 1:** Comparison of baseline variables between respondents who participated at follow-up and those who did not

Characteristic	Completers		Non-completers		*p*-Value^a^
	M (SD)/%	*N*	M (SD)/%	*N*	
Age	40.2 (12.8)	309	40.3 (13.4)	89	0.95
Gender (%)					
Male	39.0	121	44.3	39	0.37
Female	61.0	189	55.7	49	
Ethnic background (%)					
White British	53.1	164	56.2	50	0.64
White Other	11.0	34	6.7	6	
Black/Black British	19.1	59	23.6	21	
Asian/Asian British	9.7	30	7.9	7	
Mixed	7.1	22	5.6	5	
Born in the UK (%)					
No	23.6	72	19.3	17	0.40
Yes	76.4	233	80.7	71	
Housing (%)					
Independent accommodation	91.9	284	82.0	73	**0.01**
Other	8.1	25	18.0	16	
Contact with children under 16 (%)					
Living with dependent children	16.5	51	18.0	16	0.73
Other	83.6	259	82.0	73	
Education attainment (%)					
No qualifications	17.5	54	24.7	22	0.18
Other qualifications	53.4	165	53.9	48	
Degree	29.1	90	21.4	19	
Employment (%)					
No	60.3	187	78.7	70	**0.01**
In voluntary, protected or sheltered work	9.0	28	5.6	5	
In regular employment	30.7	95	15.7	14	
Living with a partner or with family (%)					
No	55.7	172	46.1	41	0.11
Yes	44.3	137	53.9	48	
Loneliness (range 8–32)	21.9 (4.9)	310	21.7 (5.4)	89	0.68
Social network size (range 0–10)	4.9 (2.3)	310	4.8 (2.2)	89	0.55
Social capital (range − 6 − 6)	2.6 (2.8)	308	2.2 (3.4)	88	0.28
Number of psychiatric inpatient hospitalisations (%)					
Never	37.1	115	37.1	33	0.37
Once	21.9	68	20.2	18	
2–5 times	26.8	83	21.4	19	
More than 5 times	14.2	44	21.4	19	
Number of years since first contact with mental health services (%)					
Less than 3 months	18.1	56	12.5	11	0.57
3 months – 1 year	9.0	28	12.5	11	
1–2 years	6.8	21	8.0	7	
2–10 years	30.7	95	35.2	31	
More than 10 years	35.5	110	31.8	28	
Diagnosis (%)					
Psychosis	25.2	77	33.7	29	0.31
Bipolar affective disorder/Manic episode	16.7	51	15.1	13	
Depressive/Anxiety disorders	37.3	114	26.7	23	
Personality disorders	12.4	38	16.3	14	
Other disorders	8.5	26	8.1	7	
Overall symptom severity (range 24–168)	43.5 (11.5)	309	45.1 (12.9)	84	0.29
Affective symptoms (range 4–28)	12.5 (5.5)	310	12.7 (6.4)	89	0.80
Self-rated recovery (range 0–88)	51.8 (17.0)	308	50.4 (18.6)	89	0.52
Health-related quality of life (range 0–100)	52.9 (23.6)	309	53.1 (26.0)	87	0.96

*M* mean, *SD* standard deviation, *N* number of participants

For instruments (loneliness, social network size, social capital, overall symptom severity, affective symptoms, self-rated recovery, health-related quality of life) range of scores is indicated between brackets

^a^Significant *p*-values printed in bold

### Descriptive results of outcomes at 4-month follow-up

The median and IQR of the four outcomes are reported due to their slightly skewed total scores. The median overall symptom severity score (range 24–168) at 4-month follow-up was 37 (IQR 30–48), the median affective symptoms score (range 4–28) was 10 (IQR 6–15), the median for self-rated recovery (range 0–88) was 59 (IQR 47–66), and the median for health-related quality of life (range 0–100) was 60 (IQR 50–76).

These four outcomes significantly improved from baseline to 4-month follow-up (Additional file 1: Table S1). Overall symptom severity (mean (SD) 43.6 (11.5) vs 40.0 (11.9), *p* < 0.001) and affective symptoms (mean (SD) 12.5 (5.5) vs 10.8 (5.3), *p* < 0.001) decreased between the two time points, while self-rated recovery (mean (SD) 51.8 (17.0) vs 56.8 (15.9), *p* < 0.001) and health related quality of life (mean (SD) 53.1 (23.7) vs 59.6 (21.7), *p* < 0.001) increased. However, the effect size of their changes was small (Cohen’s d − 0.29 – 0.33).

### Association between loneliness at baseline and outcomes at follow-up

For overall symptom severity, univariate linear regression analysis showed that greater baseline loneliness was significantly related to more severe overall symptoms at follow-up (coefficient = 0.92, 95% CI 0.66, 1.17). This association persisted after adjusting for social network size and social capital, although the association between loneliness and overall symptom severity was slightly reduced (coefficient = 0.77, 95% CI 0.50, 1.05). In model 3 where all the three blocks of independent variables (psychosocial, socio-demographic and psychiatric variables associated with both baseline loneliness and follow-up overall symptom severity with *p* < 0.25) were entered into the regression model, a 1-point higher loneliness score was associated with a 0.74-point (95% CI 0.45, 1.02) higher overall symptom severity score at follow-up. The model explained 18.1% of the variance in overall symptom severity. However, when baseline overall symptom severity score was considered simultaneously in model 4, only baseline symptom severity predicted overall symptoms at 4-month follow-up, and the association with loneliness became borderline significant (coefficient = 0.25, 95% CI − 0.03, 0.53). The final model explained 34.4% of the variance in overall symptom severity. The results of the four models with overall symptom severity as the outcome are presented in Table 2.

**Table 2:** Potential risk factors of severe overall symptoms at 4-month follow-up^a^

Variables	Model 1			Model 2			Model 3			Model 4		
	Coefficient	95% CI	*p*-Value^b^	Coefficient^c^	95% CI	*p*-Value	Coefficient	95% CI	*p*-Value	Coefficient	95% CI	*p*-Value
**Psychosocial variables**												
Loneliness (ULS-8)	0.92	0.66, 1.17	**< 0.001**	0.77	0.50, 1.05	**< 0.001**	0.74	0.45, 1.02	**< 0.001**	0.25	-0.03, 0.53	0.08
Social network size (2 items from LSNS-6)				−0.37	−0.95, 0.22	0.22	−0.34	−0.95, 0.27	0.27	−0.36	−0.91, 0.19	0.20
Social capital (HLSSC)				−0.49	− 0.97, − 0.02	**0.04**	−0.29	− 0.78, 0.20	0.25	− 0.08	− 0.53, 0.36	0.71
**Socio-demographic variables**												
Born in the UK							1.78	−1.32, 4.89	0.26	2.59	− 0.20, 5.39	0.07
Independent accommodation							−2.92	−7.70, 1.87	0.23	−0.15	−4.50, 4.19	0.95
Employment												
No							reference					
In voluntary, protected or sheltered work							−0.85	−5.29, 3.60	0.71	0.21	−3.78, 4.21	0.92
In regular employment							−3.81	−6.79, −0.82	**0.01**	−2.65	−5.35, 0.05	0.06
Living with a partner or with family							−0.61	−3.36, 2.13	0.66	−0.38	−2.86, 2.10	0.76
**Psychiatric variables**												
Number of years since first contact with mental health services												
Less than 3 months							reference					
3 months – 1 year							−2.93	−8.03, 2.18	0.26	− 2.13	−6.71, 2.45	0.36
1–2 years							0.59	−5.28, 6.46	0.84	2.31	−2.97, 7.59	0.39
2–10 years							1.62	−2.26, 5.50	0.41	1.50	−1.98, 4.98	0.40
More than 10 years							0.63	−3.50, 4.77	0.76	−0.07	−3.80, 3.65	0.97
Diagnosis												
Psychosis							reference					
Bipolar affective disorder/Manic episode							−0.43	−4.47, 3.62	0.84	0.29	−3.34, 3.93	0.87
Depressive/Anxiety disorders							0.05	−3.49, 3.58	0.98	0.51	−2.66, 3.68	0.75
Personality disorders							2.13	−2.26, 6.53	0.34	1.11	−2.87, 5.10	0.58
Other disorders							−2.99	−8.10, 2.12	0.25	−2.75	−7.33, 1.84	0.24
**Overall symptoms at baseline (BPRS)**										0.49	0.38, 0.61	**< 0.001**
*R*^*2*^_*adj*_	0.138			0.149			0.181			0.344		

*CI* Confidence Interval, *ULS-8* UCLA Loneliness Scale-8, *LSNS-6* Lubben Social Network Scale-6, *HLSSC* Health and Lifestyles Survey Social Capital Questionnaire, *BPRS* Brief Psychiatric Rating Scale, *R*^*2*^_*adj*_ adjusted-R^2^

^a^Using multivariable linear regression analyses with overall symptom severity score at 4-month follow-up as dependent variable and factors with *p* < 0.25 in both Additional file 1: Tables S2 and S3 as independent variables

^b^Significant *p*-values printed in bold

^c^Negative regression coefficient = less severe overall symptoms

When affective symptoms and self-rated recovery scores at 4-month follow-up were used as dependent variables respectively (Tables 3 and 4), the associations between baseline loneliness and follow-up outcomes were similar to the above ones. In model 3 where all the three blocks of independent variables (psychosocial, socio-demographic and psychiatric variables associated with both baseline loneliness and follow-up outcomes with *p* < 0.25) were entered, per 1-point increase in baseline loneliness score there is a 0.34-point (95% CI 0.21, 0.47) increase in affective symptoms score and a 1.08-point (95% CI -1.45, − 0.71) lower recovery score at follow-up. However, in the fully adjusted model 4 where baseline affective symptoms score was also entered (Table 3), more severe affective symptoms at 4-month follow-up were only predicted by its baseline score and having a diagnosis of a personality disorder. When baseline self-rated recovery score was considered simultaneously in model 4 (Table 4), poorer recovery at 4-month follow-up was only predicted by lower baseline recovery score, 1–2 years and longer time (2–10 years) since first contact with mental health services. The associations between loneliness and the two outcomes were no longer statistically significant (coefficient = 0.11, 95% CI -0.02, 0.24; coefficient = − 0.25, 95% CI -0.63, 0.13, respectively).

**Table 3:** Potential risk factors of severe affective symptoms at 4-month follow-up^a^

Variables	Model 1			Model 2			Model 3			Model 4		
	Coefficient	95% CI	*p*-Value^b^	Coefficient^c^	95% CI	*p*-Value	Coefficient	95% CI	*p*-Value	Coefficient	95% CI	*p*-Value
**Psychosocial variables**												
Loneliness (ULS-8)	0.41	0.30, 0.53	**< 0.001**	0.37	0.24, 0.49	**< 0.001**	0.34	0.21, 0.47	**< 0.001**	0.11	−0.02, 0.24	0.10
Social network size (2 items from LSNS-6)				−0.06	− 0.32, 0.21	0.68	− 0.02	− 0.30, 0.25	0.88	− 0.06	−0.31, 0.19	0.64
Social capital (HLSSC)				−0.19	−0.40, 0.02	0.08	−0.10	− 0.32, 0.12	0.39	− 0.04	− 0.25, 0.16	0.67
**Socio-demographic variables**												
Born in the UK							0.50	−0.89, 1.89	0.48	0.52	−0.75, 1.80	0.42
Employment												
No							reference					
In voluntary, protected or sheltered work							−0.95	−2.96, 1.06	0.35	−0.06	−1.92, 1.79	0.95
In regular employment							−0.70	−2.05, 0.65	0.31	−0.82	−2.06, 0.42	0.19
Living with a partner or with family							−0.72	−1.94, 0.50	0.25	−0.76	−1.88, 0.36	0.18
**Psychiatric variables**												
Number of years since first contact with mental health services												
Less than 3 months							reference					
3 months – 1 year							−2.26	−4.56, 0.05	0.06	−1.98	−4.09, 0.14	0.07
1–2 years							0.44	−2.21, 3.09	0.75	0.78	−1.65, 3.21	0.53
2–10 years							0.03	−1.72, 1.78	0.97	−0.12	−1.73, 1.49	0.88
More than 10 years							−0.28	−2.14, 1.58	0.77	−0.48	−2.18, 1.23	0.58
Diagnosis												
Psychosis							reference					
Bipolar affective disorder/Manic episode							0.83	−0.99, 2.64	0.37	0.89	−0.78, 2.56	0.29
Depressive/Anxiety disorders							1.52	−0.06, 3.10	0.06	0.75	−0.72, 2.21	0.32
Personality disorders							3.16	1.18, 5.14	**0.002**	2.11	0.27, 3.95	**0.03**
Other disorders							0.65	−1.65, 2.96	0.58	−0.40	−2.53, 1.73	0.71
**Affective symptoms at baseline (4 items from BPRS)**										0.42	0.31, 0.53	**< 0.001**
*R*^*2*^_*adj*_	0.138			0.138			0.168			0.300		

*CI* Confidence Interval, *ULS-8* UCLA Loneliness Scale-8, *LSNS-6* Lubben Social Network Scale-6, *HLSSC* Health and Lifestyles Survey Social Capital Questionnaire, *BPRS* Brief Psychiatric Rating Scale, *R*^*2*^_*adj*_ adjusted-R^2^

^a^Using multivariable linear regression analyses with affective symptoms score at 4-month follow-up as dependent variable and factors with *p* < 0.25 in both Additional file 1: Tables S2 and S3 as independent variables

^b^Significant *p*-values printed in bold

^c^Negative regression coefficient = less severe affective symptoms

**Table 4:** Potential risk factors of poor self-rated recovery at 4-month follow-up^a^

Variables	Model 1			Model 2			Model 3			Model 4		
	Coefficient^b^	95% CI	*p*-Value^c^	Coefficient	95% CI	*p*-Value	Coefficient	95% CI	*p*-Value	Coefficient	95% CI	*p*-Value
**Psychosocial variables**												
Loneliness (ULS-8)	−1.38	− 1.71, − 1.05	**< 0.001**	−1.21	− 1.57, − 0.85	**< 0.001**	−1.08	− 1.45, − 0.71	**< 0.001**	− 0.25	−0.63, 0.13	0.19
Social network size (2 items from LSNS-6)				0.29	−0.47, 1.04	0.45	0.55	−0.24, 1.34	0.17	0.32	−0.38, 1.02	0.37
Social capital (HLSSC)				0.54	−0.07, 1.15	0.08	0.44	−0.19, 1.08	0.17	0.15	−0.42, 0.17	0.62
**Socio-demographic variables**												
Born in the UK							−1.38	−5.41, 2.64	0.50	−0.19	−3.78, 3.40	0.92
Employment												
No							reference					
In voluntary, protected or sheltered work							3.56	−2.25, 9.37	0.23	−0.80	−6.11, 4.52	0.77
In regular employment							1.05	−2.84, 4.95	0.60	1.34	−2.12, 4.79	0.45
Living with a partner or with family							−0.99	−4.49, 2.51	0.58	0.13	−3.00, 3.27	0.93
**Psychiatric variables**												
Number of years since first contact with mental health services												
Less than 3 months							reference					
3 months – 1 year							0.40	−6.26, 7.06	0.91	−1.43	−7.43, 4.57	0.64
1–2 years							−9.90	− 17.41, −2.38	**0.01**	− 10.72	− 17.40, −4.02	**0.002**
2–10 years							−8.78	−13.82, −3.74	**0.001**	− 7.84	− 12.35, − 3.33	**0.001**
More than 10 years							−4.12	−9.47, 1.23	0.13	−4.49	−9.30, 0.32	0.07
Diagnosis												
Psychosis							reference					
Bipolar affective disorder/Manic episode							0.32	−4.93, 5.56	0.91	−1.55	−6.22, 3.12	0.51
Depressive/Anxiety disorders							−1.20	−5.76, 3.36	0.60	−0.60	−4.69, 3.48	0.77
Personality disorders							−3.53	−9.18, 2.12	0.22	−3.09	−8.12, 1.93	0.23
Other disorders							3.19	−3.46, 9.84	0.35	3.97	−1.97, 9.91	0.19
**Self-rated recovery at baseline (QPR)**										0.48	0.37, 0.59	**< 0.001**
*R*^*2*^_*adj*_	0.177			0.176			0.219			0.390		

*CI* Confidence Interval, *ULS-8* UCLA Loneliness Scale-8, *LSNS-6* Lubben Social Network Scale-6, *HLSSC* Health and Lifestyles Survey Social Capital Questionnaire, *QPR* Questionnaire on the Process of Recovery, *R*^*2*^_*adj*_ adjusted-R^2^

^a^Using multivariable linear regression analyses with self-rated recovery score at 4-month follow-up as dependent variable and factors with *p* < 0.25 in both Additional file 1: Tables S2 and S3 as independent variables

^b^Negative regression coefficient = poorer self-rated recovery

^c^Significant *p*-values printed in bold

In the univariate linear regression model, greater loneliness at baseline was predictive of poorer health-related quality of life at follow-up (coefficient = − 1.69, 95% CI -2.16, − 1.23) (Table 5). The association remained significant when social network size and social capital were adjusted for in model 2 (coefficient = − 1.38, 95% CI -1.88, − 0.87). After entering the three blocks of psychosocial, socio-demographic and psychiatric variables into model 3, loneliness was still a significant predictor of quality of life at follow-up. Per 1-point increase in baseline loneliness score there is a 1.27-point (95% CI -1.79, − 0.75) decrease in health-related quality of life score at follow-up. The model explained 20.7% of the variance in quality of life. In the final model, this association persisted when baseline quality of life score was considered simultaneously in model 4. Poorer quality of life at 4-month follow-up was predicted by greater loneliness (coefficient = − 0.76, 95% CI -1.31, − 0.20), and by 1–2 years and 2–10 years since first contact with mental health services. Better quality of life at follow-up, meanwhile, was predicted by larger social network size, having an “other disorder” diagnosis, and higher baseline quality of life score. The explained adjusted variance for EQ-VAS was 25.6%.

**Table 5:** Potential risk factors of poor health-related quality of life at 4-month follow-up^a^

Variables	Model 1			Model 2			Model 3			Model 4		
	Coefficient^b^	95% CI	*p*-Value^c^	Coefficient	95% CI	*p*-Value	Coefficient	95% CI	*p*-Value	Coefficient	95% CI	*p*-Value
**Psychosocial variables**												
Loneliness (ULS-8)	−1.69	− 2.16, − 1.23	**< 0.001**	−1.38	− 1.88, − 0.87	**< 0.001**	−1.27	− 1.79, − 0.75	**< 0.001**	− 0.76	−1.31, − 0.20	**0.01**
Social network size (2 items from LSNS-6)				1.04	−0.02, 2.10	0.06	1.27	0.16, 2.38	**0.03**	1.23	0.15, 2.30	**0.03**
Social capital (HLSSC)				0.80	−0.05, 1.65	0.07	0.63	−0.25, 1.52	0.16	0.39	−0.47, 1.25	0.37
**Socio-demographic variables**												
Born in the UK							−3.28	−8.92, 2.37	0.25	−3.42	− 8.91, 2.07	0.22
Employment												
No							reference					
In voluntary, protected or sheltered work							1.97	−6.21, 10.15	0.64	−0.30	−8.30, 7.70	0.94
In regular employment							2.49	−2.98, 7.96	0.37	2.09	−3.25, 7.43	0.44
Living with a partner or with family							−0.03	−4.93, 4.88	0.99	−0.31	−5.07, 4.45	0.90
**Psychiatric variables**												
Number of years since first contact with mental health services												
Less than 3 months							reference					
3 months – 1 year							6.23	−3.03, 15.49	0.19	6.33	−2.64, 15.31	0.17
1–2 years							−10.15	−20.62, 0.31	0.06	−10.21	−20.36, −0.06	**0.049**
2–10 years							−8.79	−15.87, −1.70	**0.02**	−9.01	−15.90, −2.13	**0.01**
More than 10 years							−6.68	−14.15, 0.78	0.08	−6.43	−13.66, 0.81	0.08
Diagnosis												
Psychosis							reference					
Bipolar affective disorder/Manic episode							−0.07	−7.35, 7.22	0.99	1.80	−5.33, 8.92	0.62
Depressive/Anxiety disorders							−2.23	−8.60, 4.14	0.49	−0.58	−6.82, 5.67	0.86
Personality disorders							0.65	−7.23, 8.54	0.87	3.02	−4.70, 10.74	0.44
Other disorders							9.08	−0.11, 18.27	0.05	10.18	1.25, 19.11	**0.03**
**Health-related quality of life at baseline (EQ VAS)**										0.24	0.13, 0.35	**< 0.001**
*R*^*2*^_*adj*_	0.143			0.158			0.207			0.256		

*CI* Confidence Interval, *ULS-8* UCLA Loneliness Scale-8, *LSNS-6* Lubben Social Network Scale-6, *HLSSC* Health and Lifestyles Survey Social Capital Questionnaire, *EQ VAS* EuroQol Health Questionnaire visual analogue scale, *R*^*2*^_*adj*_ adjusted-R^2^

^a^Using multivariable linear regression analyses with health-related quality of life score at 4-month follow-up as dependent variable and factors with *p* < 0.25 in both Additional file 1: Tables S2 and S3 as independent variables

^b^Negative regression coefficient = poorer health-related quality of life

^c^Significant *p*-values printed in bold

## Discussion

### Loneliness and outcomes of mental health problems

Initially, the study found that greater loneliness at baseline predicted more severe overall symptoms and affective symptoms, and poorer self-rated recovery and health-related quality of life at 4-month follow-up. These associations were independent of social network size and social capital, and persisted with adjustment for socio-demographic and clinical potential confounders. Loneliness seems to be a better predictor of clinical outcomes and quality of life than objective social isolation, i.e. social network size and living with a partner or with family, and neighbourhood social capital, none of which predicted overall symptom severity, affective symptoms or self-rated recovery with loneliness also in the model. In health-related quality of life, both loneliness and social network size were predictive of the outcome, but the standardised regression coefficient of loneliness was larger than the coefficient of social network size (beta − 0.29 vs 0.13). The results cohere with the finding of an existing systematic review that perceived quality of social relationships was more closely associated with depression in older adults than quantitative aspects of social relationships [1].

However, associations between baseline loneliness and follow-up scores for overall symptom severity, affective symptoms and self-rated recovery were no longer statistically significant (*p* < 0.05) once adjustment was made for baseline values for the three outcomes. This is difficult to interpret clearly, as the extent of change in outcomes between our baseline and the 4 month follow-up was relatively small, with Cohen’s *d* around 0.3 (lower than 0.41 which has been proposed as a threshold for a “practically” significant effect [48]). Since the outcomes only slightly changed from baseline to follow up, it is perhaps unsurprising that when baseline outcome variable scores were added to the regression models, loneliness did not remain significant. Similarly, a study among adults with anxiety or depressive disorder reported that greater loneliness at baseline predicted more severe anxiety symptoms at 1-year follow-up in univariate analysis but not in multivariable models where the outcome measure at baseline was controlled for, although loneliness remained to be a predictor of depressive symptom severity in the fully adjusted model [49]. In another research of depression in late life, however, baseline loneliness remained a significant determinant of depressive symptom severity at 2-year follow-up after adjustment for the outcome measure at baseline [50]. Our study illustrates the long-identified dilemma about whether to adjust for uncontrolled baseline differences in outcome between groups [51] – i.e. in our study, differences on health outcome measures at baseline between more and less lonely participants. Potential explanations for lack of consistency in finding an association between baseline loneliness and outcome include: i) There is evidence of a strong relationship between loneliness and symptoms/recovery, but the direction of effect may be that people with more severe symptoms or who have recovered less well feel more lonely rather than vice versa; (ii) it may be that by the pre-baseline point, loneliness has already had an effect on these health outcomes, and that this effect persists but does not increase through the follow-up period; (iii) in our study, it may be that the baseline measurement occurred after the main changes in outcomes that followed the crisis had already occurred (between CRT admission and our baseline at CRT discharge), reflected in only small changes on the outcome measures over our study period.

In terms of health-related quality of life, greater loneliness at baseline significantly predicted the poor outcome at follow-up even after the baseline measure of quality of life was controlled for. The finding is consistent with a cross-sectional study of late-life depression which reported that severe loneliness was more closely associated with poorer quality of life than no/mild loneliness [52]. However, no other studies have longitudinally examined the association between loneliness and quality of life in mental health service users. Since loneliness is closely related to perceived social support, an existing longitudinal study [53] of perceived social support and quality of life in people with psychosis and mood disorders is relevant. The authors reported that greater perceived social support was a significant determinant of better quality of life at 18 months [53]. Thus the evidence so far suggests that subjective appraisal of social relationships influences quality of life outcomes.

### Implications

Our study found a clear longitudinal relationship between loneliness and health-related quality of life, and cross-sectional evidence of relationships with symptoms and personal recovery, but with the direction for causality not clearly established. In order to fully understand the relationship between loneliness and mental health outcomes, it is necessary for future cohort studies to have a longer follow-up period and multiple time points. In addition, investigating the mechanisms through which loneliness impact mental health is helpful to understand reasons why people feel lonely and inform the development of interventions. The mechanisms by which loneliness affects mental health are, by and large, far from clear. Researchers need to disentangle whether loneliness shares the same pathways with other social relationships or possesses its specific pathways. Some psychosocial difficulties and personal qualities that have not been included in our study may affect both mental health and loneliness. Internalised stigma, poor interpersonal competence and low self-esteem are a common feature in many mental health problems and have been reported to be closely related to loneliness among service users [54–56]. Apart from these psychosocial factors, there is another debate about whether social relationships affect health at all times (main effects) or only when a person encounters stress or other health risks (buffering effects) [57]. If the main and buffering effects share the same mechanism, there may be a greater impact of loneliness on mental health when a person confronts stress or other health risks. Alternatively, there may be a variety of mechanisms underlying buffering and main effects. A study of the general population confirmed that perceived stress mediated the association between loneliness and general health [58]. However, the evidence in mental health fields is scarce. House et al. [57] suggested that “the interrelationships among multiple social, psychological, behavioural, and biological processes and mechanisms” need to be assessed and studied simultaneously to promote our knowledge of these issues. Future longitudinal studies with a longer follow-up period and multiple time points could examine i) biological (e.g. brain activation), psychological (e.g. internalised stigma) and/or behavioural (e.g. healthy behaviours) factors as mediation mechanisms in the association of loneliness with follow-up mental health outcomes, and ii) potential stress differences in the predictive associations of loneliness with mental health outcomes at follow-up.

As loneliness was reported to be a better predictor of mental health outcomes than objective social isolation in both our study and previous research [1], we suggest that mental health professionals assess perceived quality of social relationships, especially loneliness, not just quantitative aspects of social relationships. In addition to screening for loneliness, it may be helpful for practitioners to have clinical conversations with service users about their perceptions of causes of loneliness and possible solutions for that individual, considering that a marked decrease in loneliness may have positive impact on mental health service users’ recovery. Although there is no robust evidence base to guide clinicians in how best to mitigate loneliness, individualised interventions may nevertheless be helpful for clients who experience severe loneliness [59]. The individualised interventions could be agreed collaboratively and reviewed regularly to check whether they are acceptable and experienced as helpful. On balance, a personalised response to loneliness is important, considering the nature of loneliness as perceived deficiencies in one’s social relationships. It is also important to develop and get more evidence on loneliness interventions for people who experience mental health crises.

Furthermore, efforts can be made at the policy level. Determinants of social relationships exist on multiple levels, however current loneliness interventions do not have enough focus on macrosocial determinants of loneliness [59]. Poverty and inequality are worldwide issues and they are associated with greater loneliness [60, 61] and mental health problems [62]. In our sample, over 60% of participants had no employment and around 20% had no qualifications. Public policy to attenuate deprivation and boost employment, education and housing opportunities might prevent chronic loneliness from being established. Social, political and economic improvement should be made to decrease their powerful negative effect on both people’s health and its social determinants [63].

## Strengths and limitations

An important strength of the study concerns the longitudinal impact of loneliness among CRT users. The sample represented a full spectrum of people with relatively severe mental disorders which needed mental health crisis care. The study offered preliminary evidence, not previously available, about the impact of loneliness on recovery among people following mental health crises. A greater precision of the effect estimates was guaranteed by the large sample size, standardised procedures, repeated assessment, and the use of well-validated scales. Moreover, the results were obtained on the basis of rigorous statistical analyses, e.g. the outcome measures at baseline were consistently controlled, thereby increasing precision of the findings.

However, several limitations of the study are worthy of note. In terms of duration of follow-up, the 4-month follow-up period was not very long to identify clear longitudinal relationships: we chose this period anticipating significant change as participants recovered from crises, but this expectation was not fulfilled. In the previous two studies where baseline loneliness significantly predicted depressive symptoms in the fully adjusted models, participants were followed up more than 1 year and there were marked changes between baseline and follow-up depressive symptoms [49, 50].

Another limitation is the loss at follow-up of 22.3% of baseline participants, although the attrition rate is comparable to that for similar study populations [49, 64]. Furthermore, significant differences between follow-up completers and non-completers only existed in housing and employment situation. Participants who had independent accommodation and those who had regular employment or voluntary, protected or sheltered work were more likely to take part in the follow-up assessment. However, neither of them was related to loneliness or outcome measures in multivariable analyses. In addition, there may be an interest bias in our sample as people in our study were willing to participate in a trial involving peer support. The exclusion of four items from LSNS-6 is another limitation. It might change psychometric properties of the scale and make it difficult to compare these scores against results of other studies.

Finally, the strength of associations between loneliness and outcomes in mental disorders might differ by psychiatric diagnoses. This paper is unable to explore their associations separately due to the limited sample size of each diagnosis. However, CRTs, as a type of community crisis service, involve users with a wide range of diagnoses all of whom are immediately post mental health crisis. It offers a genuine baseline with everyone starting from a shared experience of acute service use. It is also a good group for researchers to begin to understand the impact of loneliness on outcomes in relatively severe mental health problems.

## Conclusions

The quantitative investigation in this study found an association between greater loneliness at baseline and poorer health-related quality of life at follow-up, and that loneliness seems to be a better predictor of overall symptom severity, affective symptoms and self-rated recovery than objective social isolation and neighbourhood social capital. However, the associations between loneliness and clinical outcomes were significantly attenuated by controlling for their baseline values, with ambiguity about how this should be interpreted. These findings support an opinion that loneliness could be a promising target to improve recovery for people with mental health problems. The study also implicates that public awareness of the health hazard posed by loneliness should be advanced, and that practice and policy level changes should be promoted.

## Supplementary information

**Additional file 1.** Flowchart of participants at inclusion and follow-up and supplementary results tables.

## Data Availability

The datasets used and analysed during the current study are available from the corresponding author on reasonable request.

## Abbreviations

CRT: Crisis resolution team
CORE: CRT Optimisation and RElapse prevention
NHS: National Health Service
BPRS: Brief Psychiatric Rating Scale
QPR: Questionnaire on the Process of Recovery
VAS: visual analogue scale
EQ-5D: Euroqol 5-Dimension Health Questionnaire
ULS-8: University of California at Los Angeles Loneliness Scale
LSNS: Lubben Social Network Scale
