# Supplementary material for: Loneliness as a predictor of outcomes in mental disorders among people who have experienced a mental health crisis: a 4-month prospective study
Source: BMC Psychiatry. 2020 May 20;20:249. doi: 10.1186/s12888-020-02665-2 (PMC7238641; doi:10.1186/s12888-020-02665-2)
Supplement: Supplementary file 1 — Additional file 1. Flowchart of participants at inclusion and follow-up and supplementary results tables. [file 12888_2020_2665_MOESM1_ESM.pdf]

Additional file 1: Flowchart of participants at inclusion and follow-up and supplementary results tables

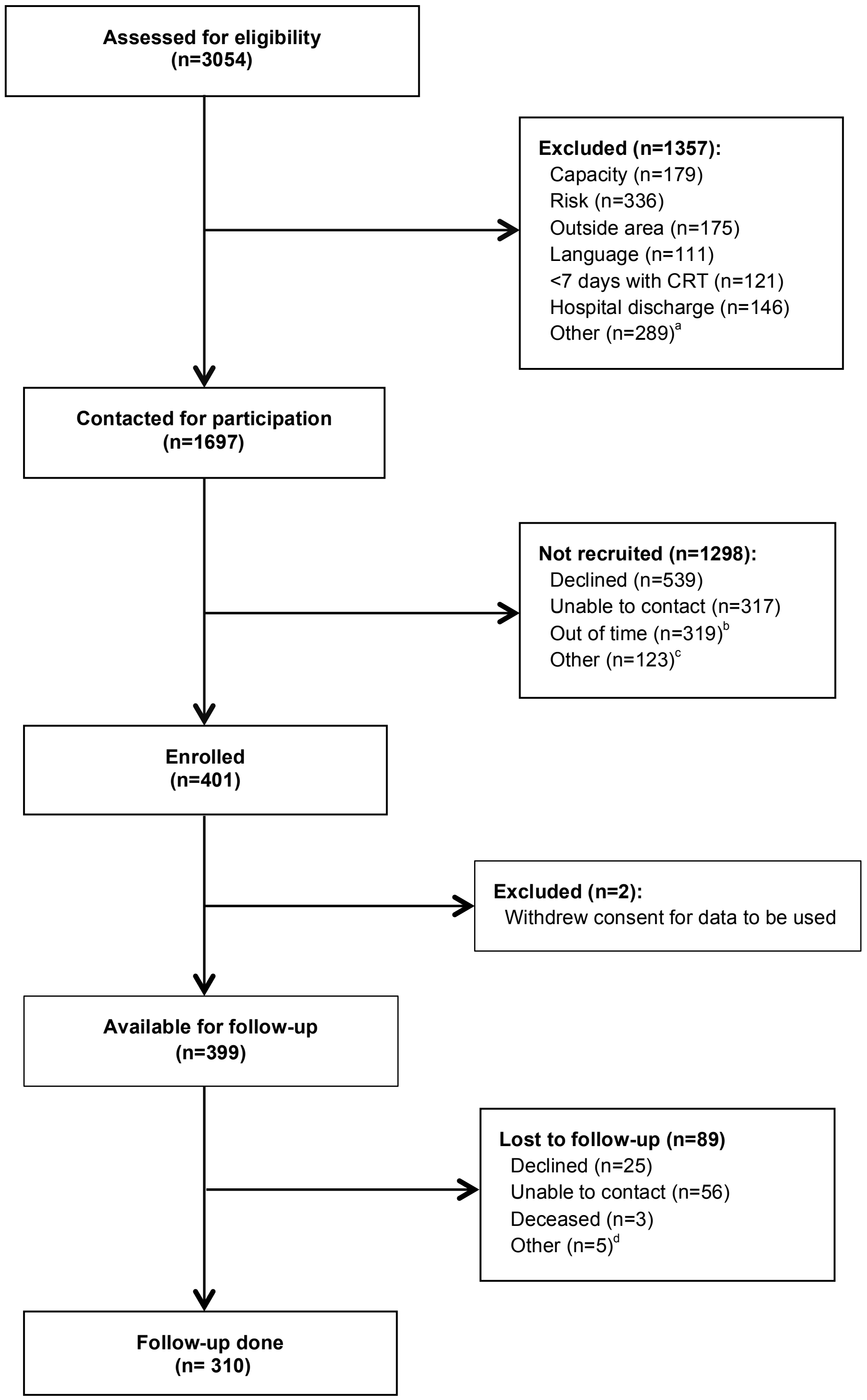

Figure S1. Flowchart of participants at inclusion and 4-month follow-up.

[a] Other = Already taking part/declined/screened; Unable to contact/engage; Amendments to screening process (recruitment of non-psychosis/bipolar participants paused April/May 2015)

[b] Out of time = researcher contact but no definitive response from potential participant within 1 month of CRT discharge

[c] Other = Temporary suspension of recruitment of non-psychosis/bipolar participants; Change in risk/capacity status since screening

[d] Other = Unwell; Risk

Table S1. Differences between outcome variables at baseline and those at 4-month follow-up

Variables	Baseline, M (SD)	Follow-up, M (SD)	*p*-Value	Effect size (Cohen's *d*)
Overall symptom severity (24−168)	43.6 (11.5)	40.0 (11.9)	<0.001	0.33
Affective symptoms (4−28)	12.5 (5.5)	10.8 (5.3)	<0.001	0.32
Self-rated recovery (0−88)	51.8 (17.0)	56.8 (15.9)	<0.001	-0.32
Health-related quality of life (0−100)	53.1 (23.7)	59.6 (21.7)	<0.001	-0.29

M, mean; SD, standard deviation.
Possible range of scores is indicated between brackets.

Table S2. Results of univariate linear regression analyses for factors associated with baseline loneliness

Variables	Coefficient[a]	95% CI	*p*-Value[b]
**Socio-demographic variables**			
Age (years)	-0.03	-0.07, 0.01	0.11
Female gender	0.39	-0.62, 1.39	0.45
Ethnic background			
White British	reference		
White Other	-0.13	-1.82, 1.57	0.88
Black/Black British	-0.44	-1.73, 0.85	0.50
Asian/Asian British	0.62	-1.13, 2.37	0.49
Mixed	-0.83	-2.84, 1.18	0.42
Born in the UK	1.13	-0.04, 2.31	0.06
Independent accommodation	-1.08	-2.70, 0.53	0.19
Living with children under 16	-0.73	-2.05, 0.58	0.27
Education attainment			
No qualifications	reference		
Other qualifications	-0.11	-1.42, 1.20	0.87
Degree	0.23	-1.23, 1.70	0.76
Employment			
No	reference		
In voluntary, protected or sheltered work	-0.25	-2.06, 1.56	0.79
In regular employment	-1.00	-2.12, 0.12	0.08
Living with a partner or with family	-0.94	-1.93, 0.04	0.06
**Psychosocial variables**			
Social network size (2 items from LSNS-6)	-0.79	-1.00, -0.59	**<0.001**
Social capital (HLSSC)	-0.52	-0.68, -0.36	**<0.001**
**Psychiatric variables**			
Number of psychiatric inpatient hospitalisations			
Never	reference		
Once	-1.29	-2.60, 0.03	0.06
2-5 times	-1.46	-2.71, -0.21	**0.02**
More than 5 times	-2.13	-3.59, -0.67	**0.004**
Number of years since first contact with mental health services			
Less than 3 months	reference		
3 months – 1 year	1.37	-0.59, 3.34	0.17
1-2 years	1.80	-0.39, 4.00	0.11
2-10 years	1.58	0.10, 3.05	**0.04**
More than 10 years	1.23	-0.23, 2.68	0.10
Diagnosis			
Psychosis	reference		
Bipolar affective disorder/Manic episode	-0.85	-2.37, 0.67	0.27
Depressive/Anxiety disorders	1.97	0.73, 3.21	**0.002**
Personality disorders	2.60	0.98, 4.23	**0.002**
Other disorders	1.93	0.01, 3.84	**0.048**

CI, Confidence Interval; LSNS-6, Lubben Social Network Scale-6; HLSSC, Health and Lifestyles Survey Social Capital Questionnaire.

[a]Negative regression coefficient = less loneliness

[b]Significant *p*-values printed in bold.

Table S3. Results of univariate linear regression analyses for baseline factors associated with outcomes at 4-month follow-up

Variables	Overall symptoms[a]	Affective symptoms[b]	Self-rated recovery[c]	Health-related quality of life[d]
	Regression coefficients (95% CI)			
**Socio-demographic variables**				
Age (years)	0.04 (-0.06, 0.15)	0.01 (-0.03, 0.06)	0.03 (-0.11, 0.17)	-0.05 (-0.24, 0.14)
Female gender	2.17* (-0.57, 4.91)	1.09* (-0.14, 2.32)	-0.44 (-4.08, 3.19)	-3.42* (-8.44, 1.59)
Ethnic background				
White British	reference			
White Other	-3.40* (-7.83, 1.04)	-1.64* (-3.62, 0.35)	3.66* (-2.24, 9.56)	4.78 (-3.52, 13.08)
Black/Black British	0.78 (-2.80, 4.36)	-0.51 (-2.11, 1.09)	2.41 (-2.34, 7.16)	1.27 (-5.29, 7.84)
Asian/Asian British	-1.21 (-5.88, 3.46)	-1.44* (-3.53, 0.65)	-0.67 (-6.89, 5.54)	0.50 (-8.03, 9.03)
Mixed	-0.02 (-5.36, 5.32)	-1.80* (-4.18, 0.59)	3.02 (-4.08, 10.13)	-1.51 (-11.26, 8.23)
Born in the UK	2.86* (-0.30, 6.01)	1.21* (-0.20, 2.63)	-2.95* (-7.16, 1.25)	-4.22* (-10.04, 1.61)
Independent accommodation	-4.73* (-9.69, 0.24)	-0.88 (-3.13, 1.36)	1.93 (-4.58, 8.45)	3.88 (-5.01, 12.78)
Living with children under 16	-1.14 (-4.73, 2.46)	-1.14* (-2.75, 0.47)	1.07 (-3.72, 5.85)	3.82 (-2.72, 10.36)
Education attainment				
No qualifications	reference			
Other qualifications	-3.07* (-6.71, 0.57)	-0.52 (-2.18, 1.13)	2.23 (-2.62, 7.07)	1.78 (-4.87, 8.42)
Degree	-3.88* (-7.87, 0.11)	-0.42 (-2.24, 1.40)	1.81 (-3.51, 7.13)	-1.92 (-9.25, 5.40)
Employment				
No	reference			
In voluntary, protected or sheltered work	-1.97 (-6.61, 2.67)	-1.80* (-3.92, 0.32)	4.60* (-1.68, 10.88)	4.88 (-3.84, 13.59)
In regular employment	-6.03** (-8.93, -3.13)	-1.41** (-2.73, -0.09)	4.44** (0.53, 8.34)	7.41** (2.04, 12.77)
Living with a partner or with family	-3.50** (-6.16, -0.84)	-1.47** (-2.67, -0.28)	2.51* (-1.06, 6.08)	4.90* (-0.02, 9.82)
**Psychosocial variables**				
Loneliness (ULS-8)	0.92** (0.66, 1.17)	0.41** (0.30, 0.53)	-1.38** (-1.71, -1.05)	-1.69** (-2.16, -1.23)
Social network size (2 items from LSNS-6)	-1.04** (-1.62, -0.46)	-0.37** (-0.63, -0.11)	1.32** (0.55, 2.08)	2.29** (1.25, 3.34)
Social capital (HLSSC)	-0.97** (-1.44, -0.50)	-0.39** (-0.60, -0.18)	1.24** (0.62, 1.86)	1.74** (0.89, 2.59)

Variables	Overall symptoms[a]	Affective symptoms[b]	Self-rated recovery[c]	Health-related quality of life[d]
	Regression coefficients (95% CI)			
(Continued from previous page)				
**Psychiatric variables**				
Number of psychiatric inpatient hospitalisations				
Never	reference			
Once	-0.30 (-3.94, 3.33)	-0.27 (-1.89, 1.36)	-1.96 (-6.74, 2.81)	-0.76 (-7.38, 5.86)
2-5 times	1.45 (-1.95, 4.86)	-0.53 (-2.06, 1.00)	-2.02 (-6.51, 2.48)	-2.92 (-9.13, 3.28)
More than 5 times	1.93 (-2.24, 6.10)	-0.50 (-2.37, 1.38)	2.14 (-3.39, 7.67)	-2.69 (-10.38, 4.99)
Number of years since first contact with mental health services				
Less than 3 months	reference			
3 months – 1 year	-1.27 (-6.65, 4.11)	-1.84* (-4.26, 0.58)	-0.27 (-7.26, 6.72)	3.77 (-5.87, 13.41)
1-2 years	5.63* (-0.53, 11.80)	2.30* (-0.42, 5.02)	-13.05** (-20.78, -5.32)	-15.53** (-26.17, -4.89)
2-10 years	4.20** (0.28, 8.13)	1.00 (-0.77, 2.77)	-10.27** (-15.36, -5.18)	-11.34** (-18.45, -4.23)
More than 10 years	4.17** (0.34, 8.01)	0.79 (-0.93, 2.52)	-5.64** (-10.60, -0.68)	-10.33** (-17.20, -3.45)
Diagnosis				
Psychosis	reference			
Bipolar affective disorder/Manic episode	-2.46 (-6.67, 1.74)	0.13 (-1.74, 1.99)	0.49 (-5.12, 6.09)	1.52 (-6.20, 9.25)
Depressive/Anxiety disorders	-0.90 (-4.35, 2.56)	1.48* (-0.05, 3.01)	-2.82* (-7.40, 1.75)	-1.53 (-7.85, 4.79)
Personality disorders	4.00* (-0.65, 8.66)	3.98** (1.92, 6.04)	-7.35** (-13.50, -1.20)	-3.61 (-12.12, 4.90)
Other disorders	-3.23* (-8.51, 2.05)	0.61 (-1.73, 2.95)	3.05 (-3.99, 10.08)	9.82** (0.17, 19.47)

ULS-8, UCLA Loneliness Scale-8; LSNS-6, Lubben Social Network Scale-6; HLSSC, Health and Lifestyles Survey Social Capital Questionnaire.

[a]Using univariate linear regression analyses with overall symptom severity score at 4-month follow-up as dependent variable. Negative regression coefficient = less severe overall symptoms.

[b]Using univariate linear regression analyses with affective symptoms score at 4-month follow-up as dependent variable. Negative regression coefficient = less severe affective symptoms.

[c]Using univariate linear regression analyses with self-rated recovery score at 4-month follow-up as dependent variable. Negative regression coefficient = poorer self-rated recovery.

[d]Using univariate linear regression analyses with health-related quality of life score at 4-month follow-up as dependent variable. Negative regression coefficient = poorer health-related quality of life.

*p<0.25

**p<0.05
